# Expanding the measurement of culture with a sample of two billion humans

**DOI:** 10.1098/rsif.2022.0085

**Published:** 2022-05-25

**Authors:** Nick Obradovich, Ömer Özak, Ignacio Martín, Ignacio Ortuño-Ortín, Edmond Awad, Manuel Cebrián, Rubén Cuevas, Klaus Desmet, Iyad Rahwan, Ángel Cuevas

**Affiliations:** ^1^ Center for Humans and Machines, Max Planck Institute for Human Development, 14195 Berlin, Germany; ^2^ Department of Economics and Center for Scientific Computing, Southern Methodist University, Dallas, TX 75205, USA; ^3^ IZA, Institute of Labor Economics, 53113 Bonn, Germany; ^4^ GLO, Global Labor Organization, 45141 Essen, Germany; ^5^ Nommon Solutions and Technologies, 28020 Madrid, Spain; ^6^ Department of Telematic Engineering, Universidad Carlos III de Madrid, 28911 Leganés (Madrid), Spain; ^7^ Department of Economics,Universidad Carlos III de Madrid, 28903 Getafe (Madrid), Spain; ^8^ Department of Economics, University of Exeter Business School, Exeter EX4 4PU, UK; ^9^ Department of Telematic Engineering and UC3M-Santander Big Data Institute, Universidad Carlos III de Madrid, 28911 Leganés (Madrid), Spain; ^10^ Department of Economics and Cox School of Business, Southern Methodist University, Dallas, TX 75205, USA; ^11^ NBER, National Bureau of Economic Research, Cambridge, MA 02138, USA; ^12^ CEPR, Centre for Economic Policy Research, London EC1V 0DX, UK

**Keywords:** culture, cultural distance, identity, regional culture, gender differences

## Abstract

Culture has played a pivotal role in human evolution. Yet, the ability of social scientists to study culture is limited by the currently available measurement instruments. Scholars of culture must regularly choose between scalable but sparse survey-based methods or restricted but rich ethnographic methods. Here, we demonstrate that massive online social networks can advance the study of human culture by providing quantitative, scalable and high-resolution measurement of behaviourally revealed cultural values and preferences. We employ data across nearly 60 000 topic dimensions drawn from two billion Facebook users across 225 countries and territories. We first validate that cultural distances calculated from this measurement instrument correspond to traditional survey-based and objective measures of cross-national cultural differences. We then demonstrate that this expanded measure enables rich insight into the cultural landscape globally at previously impossible resolution. We analyse the importance of national borders in shaping culture and compare subnational divisiveness with gender divisiveness across countries. Our measure enables detailed investigation into the geopolitical stability of countries, social cleavages within small- and large-scale human groups, the integration of migrant populations and the disaffection of certain population groups from the political process, among myriad other potential future applications.

## 1. Introduction

Culture has played a pivotal role in human evolution [1–4]. As a result, the study of human culture is one of the core endeavours of the social sciences. Tens of thousands of scientists around the world study culture [5], with disciplines ranging from anthropology [6,7] to sociology [8], from political science [9–11] to economics [12–14], and from psychology [15–19] to philosophy [20]. Their work has enabled the understanding of many human social, economic and political phenomena [12,21–28], and serves as a bedrock of knowledge in the social sciences.

We focus on a comprehensive and holistic concept of culture. Following a commonly used definition in the field of cultural evolution, we say that ‘[c]ulture in its broadest sense is that which is socially rather than genetically transmitted. [· · ·] In its totality, it is that which distinguishes one human group from another’ [29]. This is similar to a concept of culture defined as ‘that complex whole which includes knowledge, beliefs, arts, morals, law, customs, and any other capabilities and habits acquired by [a human] as a member of society’ [30]. Our definition is inclusive, making no explicit value judgments about which traits might be more or less significant.

The traditional quantitative approach to the study of culture has been shaped by the historical availability of data [31], often limiting the ability to measure culture comprehensively. For most of the history of the study of culture, collecting empirical data on humans has been costly, time consuming and in many instances impossible [32]. As a result, scholars often inductively distill the broad theoretical definitions of culture using a top-down approach [31], in the sense that the scholars themselves design the surveys that measure the set of cultural constructs they are interested in. Cultural dimensions that result from this process tend to be a select few salient and measurable features of human groups such as artistic and culinary practices [33], language [34,35] and literature [36], political ideologies [37] and institutions [23], and religions and religious practices [38].

Traditionally, producing a more comprehensive description of a group’s culture required ethnographers to observe individuals in the field [39]. Their approach is more bottom-up, in the sense that ethnographers spend long periods of time observing populations, with the aim of describing their culture holistically. More recently, the information age has enabled the emergence of what we might call computational ethnography [32,40]. Humans today spend an ever-increasing amount of time on devices that continuously track and record users’ interests, beliefs, preferences, behaviours, locations and interactions. By unobtrusively observing billions of users, social media firms play the role of ethnographers, but on a massive scale.

This changing information environment enables supplementing and expanding the scientific approach to the measurement of culture. Here, we propose new methods for the bottom-up measurement of culture globally. We first explore the strengths and weaknesses of traditional quantitative approaches to the measurement of culture and show their relation to our method. We then measure culture from the bottom up and examine our measure’s performance against traditional quantitative measures. Finally, we investigate cultural questions previously impossible to examine without the combined resolution and scope that our method enables. Ultimately, high-resolution granular data are essential for our understanding of many cultural phenomena, ranging from wars and the formation of identity to the integration of immigrants and the fragmentation of societies.

## 2. Measuring culture from the bottom up

### 2.1. Traditional quantitative approach to study culture

Traditional quantitative approaches to the study of culture benefit from numerous strengths. For example, these studies of culture are often relatively low in dimension and therefore readily measurable via quantitative surveys. This parsimony has enabled excellent studies of certain cultural features in highly data-constrained settings [41,42]. Traditional approaches also provide substantial face validity: they focus on many of the concepts typically associated with culture. These traditional approaches, as a result, likely encapsulate many important constituent cultural constructs. A final benefit of the parsimony of traditional approaches is that scholars from a wide variety of fields can measure and study different aspects of culture.

Yet while the traditional approach to culture has a number of benefits, it also has various weaknesses. First, a critical question when attempting to construct a broad measure of human culture from the top down is: where do we draw the line in terms of what to measure? Surely religion should be considered part of culture. But is group-level support for a football team part of culture? What about preferences for video games [43] or television shows? What about group-level appreciation of cat videos or the colour of socks that we choose to wear? A scholar may determine religion—but not preference for a television show—to be the more theoretically important constituent of culture to measure based on the argument that television preferences are not central to the human experience. However, another scholar with a differing opinion could argue that, given the amount of time humans in the developed world spend watching television [44], the choice of a particular show reflects an important implicit value of the humans who watch it. If a line about what to measure must be drawn from the top down, scholars will very likely reasonably disagree on where, precisely, it should fall. However, the very attempt to narrow cultural constructs from the top down highlights the second weakness of this measurement strategy. The justification for excluding any particular construct is necessarily endogenous to the particular culture (and cultural bias) of the scientist(s) doing the theorizing. Crucially, where to draw this line is a direct function of the cultural preferences of each scholar’s particular human group. This endogeneity problem arises for every supervised attempt to include or exclude a concept from the measurement of culture.

Third, the parsimonious nature of top-down approaches presents its own limitations. Implicitly, traditional quantitative approaches to the study of culture tend to focus on features that provide insight into differences among human groups [45–51]. Yet, human groups may be similar in many more dimensions than they are dissimilar. Top-down definitions tend to occlude these dimensions of similarity with an implicit focus on those features—the arts, language, politics, religion and distinct traditions—that differentiate human groups.

Finally, traditional quantitative measures of culture commonly rely on self-reported answers to survey questions [52] or subjective evaluations of the particular scientists conducting the study [53]. These pose measurement challenges. Questions such as: ‘Is religion important to you?’ or ‘Do you think adultery is immoral?’ pose substantial risks of social desirability bias [54] and direct observation of subjects induces risk of Hawthorne and experimenter demand effects [55], among other related methodological concerns.

### 2.2. Social media data allow measuring culture from the bottom up

**Figure 1.**
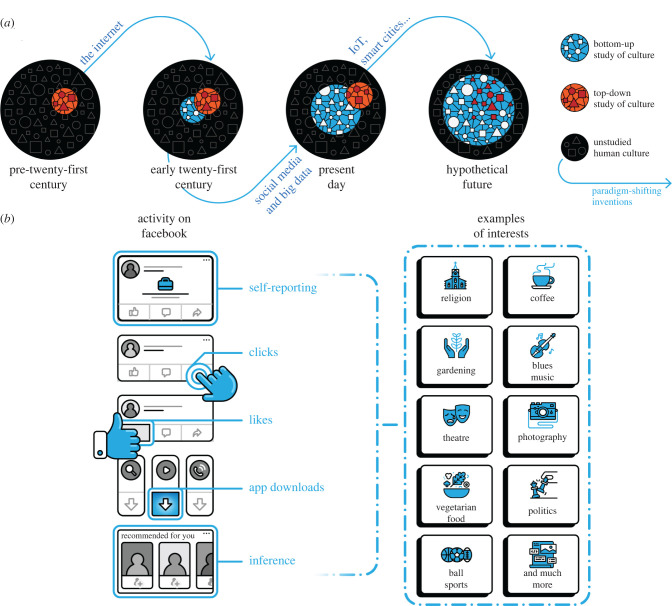
Measurement of the cultural landscape and methods of classification of values, behaviours, preferences and interests on Facebook. Panel (*a*) illustrates how the bottom-up quantitative study of culture is enabled by information technology advances and the broad measurement of humans across the globe. Paradigm shifting technologies such as the Internet, the advent of social media and big data, the introduction of the Internet of Things (IoT) and smart cities all shape the availability of information with which to measure previously unstudied dimensions of culture. We anticipate a hypothetical future in which traditional top-down concepts of culture are encompassed by and integrated into a bottom-up approach to the measurement of culture. Panel (*b*) illustrates how Facebook classifies users’ interests via users’ self-reporting, via users’ observed behaviour which includes the totality of users’ clicks on the platform and on ads served elsewhere by the platform, via users’ ‘likes’ and software downloads, and via broad inference based on users’ overall behaviour on and off the platform. The interests Facebook infers span hundreds of thousands of dimensions and include topics that both fall within more traditional measures of culture—such as religion, politics and the arts—as well as those that tend to fall outside of traditional measures—such as interests in video games, physical activity preferences and recreational drug interests.

Inspired by ethnographic methods [56], we measure culture from the bottom up, enabling a rich, unobtrusive, quantitative description of global cultural factors. We can conceive of the culture of a human group at a point in time as a complex, high-dimensional hypersurface (the black surface in figure 1*a*). This surface is not narrowed from the top down. Conceptually, it includes art and Angry Birds appreciation, ethics and email enjoyment, formalities and football fans, language and loungewear likes, religion and running routines, politics and potluck preferences, and social structures and sockwear, among every other feature of human life.

Our approach enables measuring culture from the bottom up (the blue surface in figure 1*a*), providing a complement to traditional top–down approaches (the red surface in figure 1*b*). Importantly, it prioritizes no features over others and enables the data to reveal important dimensions among observed human groups. This allows our method to be general, flexible and unsupervised. And it does not selectively exclude constructs, reducing the biases in the measurement of culture encountered by top-down methods.

But how does one go about measuring the high-dimensional hypersurface of culture? Private firms have—perhaps unintentionally—led the way. Effectively and precisely targeting products and services to individuals requires gathering massive amounts of information about those individuals’ beliefs, behaviours and preferences [57–59]. For online companies like Google and Facebook, the gathering, storing and selling of this information has itself become a multi-billion dollar enterprise. As a result, the platforms have become adroit at measuring myriad features of human lives via activities that occur during the large amount of time modern humans spend both online and offline in proximity to connected devices [60]. There is a burgeoning literature in the social sciences that relies on social media data [61–65].

Facebook places particular importance in classifying the interests of its users [66]. As a result, the company has inadvertently built the largest platform for the measurement of culture in existence (figure 1*b*). Fortunately for scholars, Facebook makes this information accessible to prospective marketers via a marketing application programming interface (API). Using information drawn from users’ self-reported interests, clicking behaviours on Facebook, likes on Facebook, software downloads, GPS location and behaviour on other sites that employ Facebook ads (figure 1*b*), this API provides the ability to create and analyse social groups of interest along hundreds of thousands of interest dimensions and down to very fine spatial and temporal resolution (the zip code-by-day level in the USA). Electronic supplementary material, appendix table B4, illustrates examples of cultural categories along with corresponding Facebook interests both for traditional and non-traditional cultural elements. By making its platform open to those interested in marketing to its users, Facebook has enabled scholars to interrogate its measures of global human interests and construct freely available measures of culture.

We use data gleaned from scraping the Facebook Marketing API to construct a high-dimensional measure of culture. We gathered nearly 60 000 diverse interests by sequentially interrogating Facebook’s platform and then constructed—for each administrative unit in our analysis—a vector of the share of individuals in that unit who held each interest. Importantly, each interest on the platform is indexed by a unique identifier, allowing for consistency across languages globally. We use these data to investigate culture at the country, subnational and local levels. Because the data we use are aggregated at the level of population groups, they cannot be used to identify any specific individual and hence do not present privacy concerns. Electronic supplementary material, appendix A, provides an in-depth discussion of Facebook’s algorithm and issues related to representativeness, biases, fake accounts and privacy.

## 3. Facebook-based distances between countries

Employing data on these interest shares drawn from over two billion individual users around the world, we first validate our measure of culture derived from Facebook interests against quantitative measures of country cultural differences taken from prior literature [12,67]. If our Facebook measure captures important components of traditional top-down measures, we expect to observe a positive correspondence between our bottom-up measure and traditional top-down measures (see electronic supplementary material, appendix A, for a description of distance and correlation measures).

**Figure 2.**
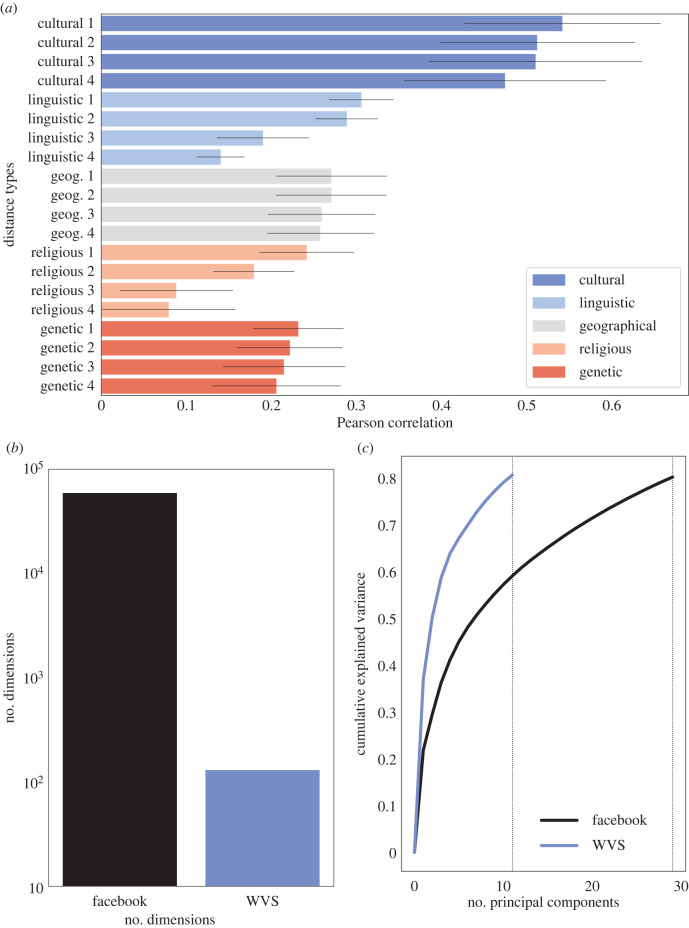
Bottom-up measurement of culture correlates with traditional top-down measures, enables the use of orders of magnitude more features, and explains additional variance. (*a*) Correlation between our bottom-up Facebook inter-country cultural distance measure and inter-country cultural distances based on traditional proxies (see electronic supplementary material, appendix A, for full list). Bars show the Pearson correlation coefficient between our measure and the four most highly correlated measures within each type of conventional proxy (cultural, linguistic, geographic, religious, and genetic). Black lines depict the 95% confidence intervals for the correlation coefficients based on Mantel tests. Results are based on the common sample of 69 countries for which all measures are available. (*b*) Bars show the number of Facebook interests and the number of common questions included in all waves of the World Values Survey (WVS). (*c*) Lines depict the number of principal components and their cumulative explained variance for each measure. To explain 80% of the variation underlying the Facebook interests data across countries 29 principal components are required, as opposed to the 11 principal components necessary to explain the same level of variation in the WVS.

Figure 2*a* presents the results of these comparisons. Our bottom-up measure of inter-country cultural distance corresponds positively and significantly to a wide variety of measures of cultural distance between countries. We observe small positive correlations between our measure and measures of linguistic, geographical, religious and genetic distance between country populations [68–77] (see electronic supplementary material, appendix A). However, between more direct measures of traditional notions of culture—provided via the World Values Survey (WVS) [52,78]—we observe a more marked positive correspondence with a correlation coefficient of approximately 0.5 (coefficient: 0.54, *p*-value: 0.0001). Thus our bottom–up measure of cultural distance corresponds positively but imperfectly to traditional measures.

Does this imperfect correspondence result from the measurement of additional components of cultural distance between countries? At face value, figure 2*b* shows that the number of Facebook interests are several orders of magnitude larger than the number of questions in the WVS. To further investigate whether this also translates into capturing more dimensions of culture, we perform principal component analysis on Facebook interests and WVS questions, using the common sample of 69 countries covered by both data sources (see electronic supplementary material, appendix A). Our goal is to reduce the dimensionality of interests and questions and to assess how many unique principal components are able to explain a large share (80%) of the variance in our Facebook measure of culture and in the WVS questions across countries.

Figure 2*c* plots the share of the overall variance in questions and interests that is explained by principal components as a function of their number. Our measure of culture derived from Facebook interests explains 80% of the variance between countries using three times the number of principal components as required to explain 80% of the variance using the WVS. This provides suggestive evidence that the Facebook measure covers a more diverse array of explanatory dimensions of culture as compared to the WVS.

While our Facebook data span a broad variety of interests, do they also capture a broader set of specific cultural traits than those measured by the WVS? To explore this question, we employ a supervised machine learning algorithm that uses all our Facebook interests to predict close to 50 specific cultural attributes, ranging from generosity to gender bias. When comparing the predicted traits to the observed traits, we find an average correlation of 0.6, indicating that the wide array of Facebook data are also able to capture specific cultural traits (see electronic supplementary material, appendix table B3).

**Figure 3.**
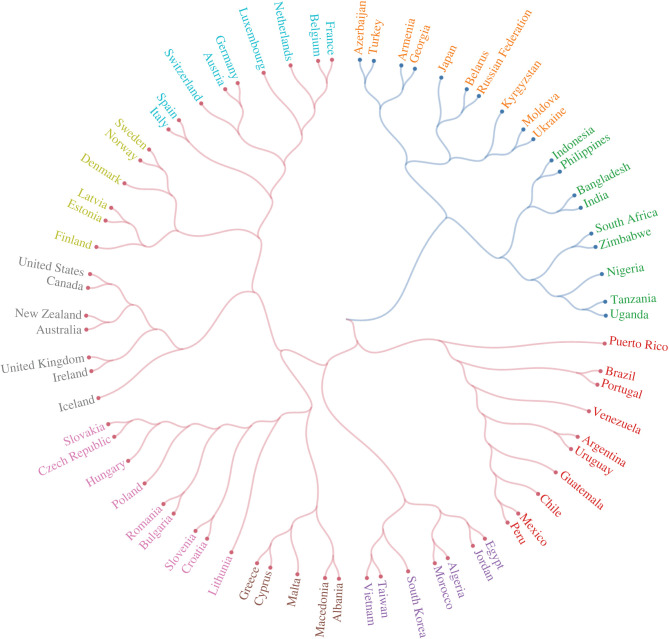
Bottom-up measurement of culture returns sensible clusters of countries. Dendrogram is generated using cosine distance and the Ward linkage method (see electronic supplementary material, appendix A). Countries and territories with at least 300 000 persons, a Facebook penetration rate of more than 5%, and representation in both the Facebook and WVS data were selected. The colour of a country’s link represents its membership of a main cluster, while the colour of its name represents its membership of a sub-cluster. Two countries of the same colour are closer to each other than to a country of a different colour.

Next, we examine whether clusters of countries returned via our Facebook measure of culture mirror common conceptions of cultural similarity, providing a measure of face validity. Figure 3 presents a dendrogram of countries, based on the cosine distance between culture vectors constructed from our Facebook data employing the Ward linkage method. The sample of countries consists of those that overlap with the WVS, have a population of more than 300 000, and have a Facebook penetration rate of more than 5% (see electronic supplementary material, appendix A). As can be seen, the unsupervised clustering of countries within our sample provides substantial validity to our measure. Countries that typically are culturally or historically associated with one another—the USA and Canada, India and Bangladesh, Germany and Austria—are placed directly next to one another in the clusters. Our approach also reveals novel features in the data that go beyond obvious geographical clustering. For example, Puerto Rico is closer to the Latin American cluster than it is to the USA, despite being a US territory. Furthermore, linguistically similar but geographically disparate countries—such as the US and Australia, and Brazil and Portugal—cluster together. It is important to mention that clustering algorithms have some difficulty in dealing with ‘outliers’. This explains the maybe surprising location of Japan in figure 3. However, when we extend the dendrogram to include all 225 countries, Japan is no longer special, appearing alongside China (see electronic supplementary material, appendix figure B16). Electronic supplementary material, appendix B, provides extensive robustness checks, exploring different ways of measuring distances and analysing different samples of countries and interests.

## 4. Facebook-based distances between subnational regions

Figures 2 and 3 provide evidence for the validity of our method. However, cultural variation is not relegated solely to nation-level groupings. Cultural differences at the subnational level are essential to understand nation-building efforts as well as geopolitical and secessionist threats around the globe. Unfortunately, traditional quantitative measures are highly costly to construct and thus provide little representative insight into subnational cultures. Conducting representative surveys at high resolution globally would be cost-prohibitive (in the limit, the costs would approach those of the Facebook platform itself). Might our Facebook measure be able to provide improved, scalable insight into novel subnational cultural variations?

**Figure 4.**
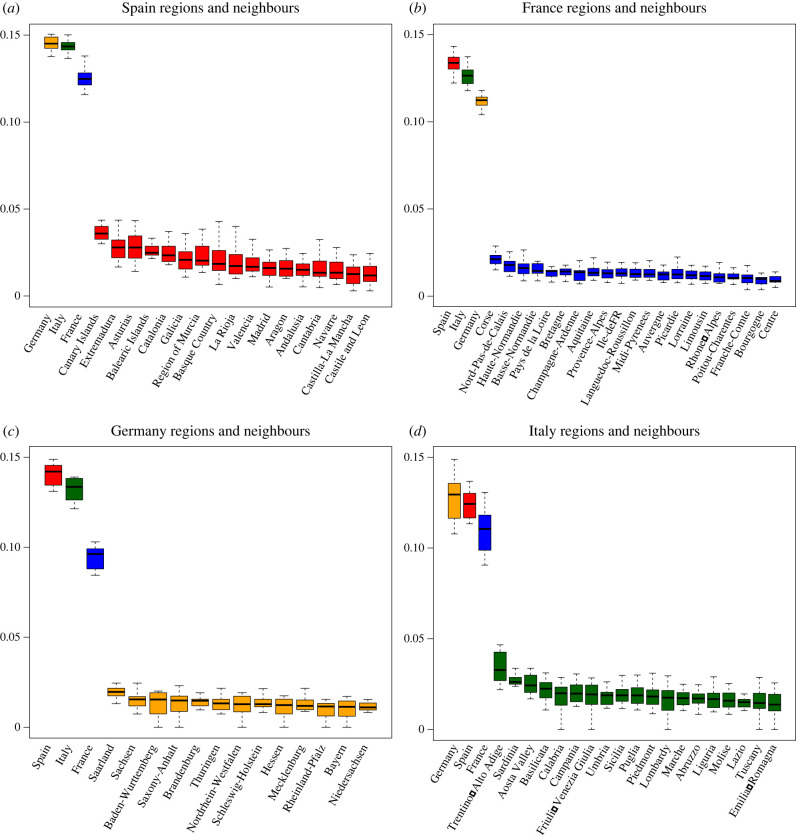
Bottom-up measurement of subnational culture indicates substantial within-country cultural similarity. (*a*) Subnational units in Spain are less distant from one another than from European cultural neighbours of Germany, Italy or France. (*b*) French regions are culturally closer to one another than to European neighbours. (*c*) German regions demonstrate greater similarity to one another than to European neighbours. (*d*) Italian regions are culturally less distant from one another than from their European neighbours. Subnational units in (*a*–*d*) are represented with boxplots that show the distance distribution between a given region and all other regions within the country under analysis. In the case of neighbouring countries, boxplots show the distance distribution between that neighbouring country and all the regions within the country under analysis. Colour coding: distances to Spain or Spanish regions (red), to France or French regions (blue), to Germany or German regions (yellow), to Italy or Italian regions (green).

To investigate this question, we gather vectors of Facebook interests for subnational regions in the USA and Europe and compute cosine distances between each region within the country and all the other regions within the same country (see electronic supplementary material, appendix A). One question is whether subnational regions are less distant from other regions within their own country than from closeby countries. Figure 4 investigates this for the regions of Spain (red), France (blue), Germany (yellow) and Italy (green). For example, panel (*a*) depicts the distribution of the cultural distances between Spanish regions, and between these regions and other European countries. The subnational regions within Spain are much less distant from one other than from neighbouring European countries. For example, Catalonia is culturally markedly closer to any other Spanish region than to either Italy or France. This same pattern holds for regions in the other countries (figure 4*b*–*d*).

Before concluding that national borders demarcate sharp cultural boundaries, we also compare cultural distances between sub-national regions in different countries. While sub-national regions are culturally closer to each other than to other countries (Paris is closer to other regions of France than to Spain), we might expect capital cities to resemble each other (Paris might be closer to Madrid than to rural regions of France). We observe the opposite. Almost all sub-regions in our data are closest to sub-regions within their own national borders. Only two sub-regions in our European data—Flanders in Belgium and Donegal County in Ireland—are closer to regions in a separate country than they are to other regions in their own nation. Both exceptions can be traced back to fairly recent changes in country borders: the splitting of the province of Limburg between Belgium and the Netherlands in the 1830s and the Partition of Ireland in the 1920s. Overall, this suggests the importance of national boundaries in shaping cultural distances. It also suggests that our measure captures deep cultural elements that persist over longer periods of time.

**Figure 5.**
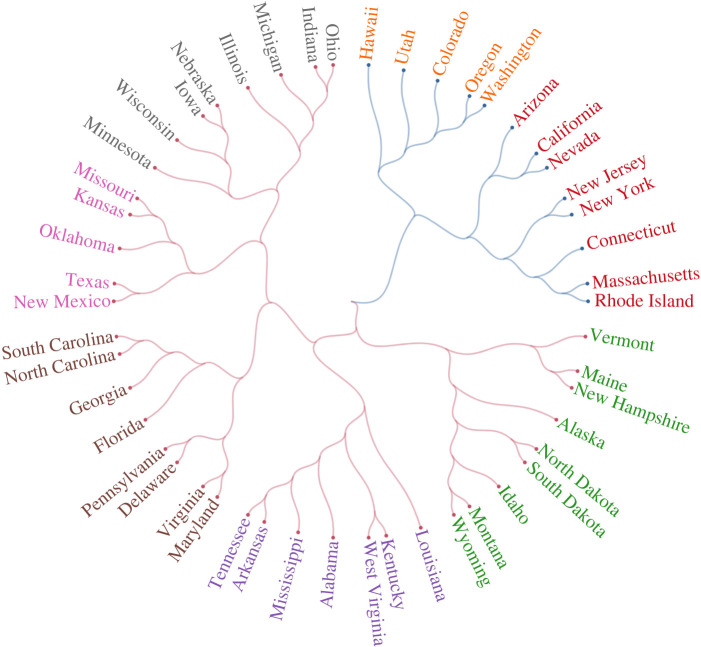
Bottom-up measurement of subnational culture returns sensible clustering of subnational units. Facebook culture vectors drawn from the states of the USA cluster together in an unsupervised manner into traditional regional and cultural groupings. The colour of a state’s link represents its membership of a main cluster, while the colour of its name represents its membership of a sub-cluster. Two states of the same colour are closer to each other than to a country of a different colour. South Carolina bears similarity to North Carolina, Montana to Wyoming, New Jersey to New York and West Virginia to Kentucky. Similarities are not strictly geographical, with Alaska bearing similarity to New Hampshire and North Dakota, for example.

Do subnational cultures as measured via our Facebook data cluster together in a sensible manner? To examine this question, we calculate cosine distances for US states and perform unsupervised clustering using the Ward linkage method (see electronic supplementary material, appendix A). Figure 5 presents the resulting dendrogram. The clusters return traditional regional and cultural groupings. For example, states in the US Midwest are placed in proximity to one another, as are the states in the US South. Mountainous and more rural states also cluster together, with Alaska being closest to states like North Dakota, Idaho and New Hampshire, despite the substantial geographical distances between them.

**Figure 6.**
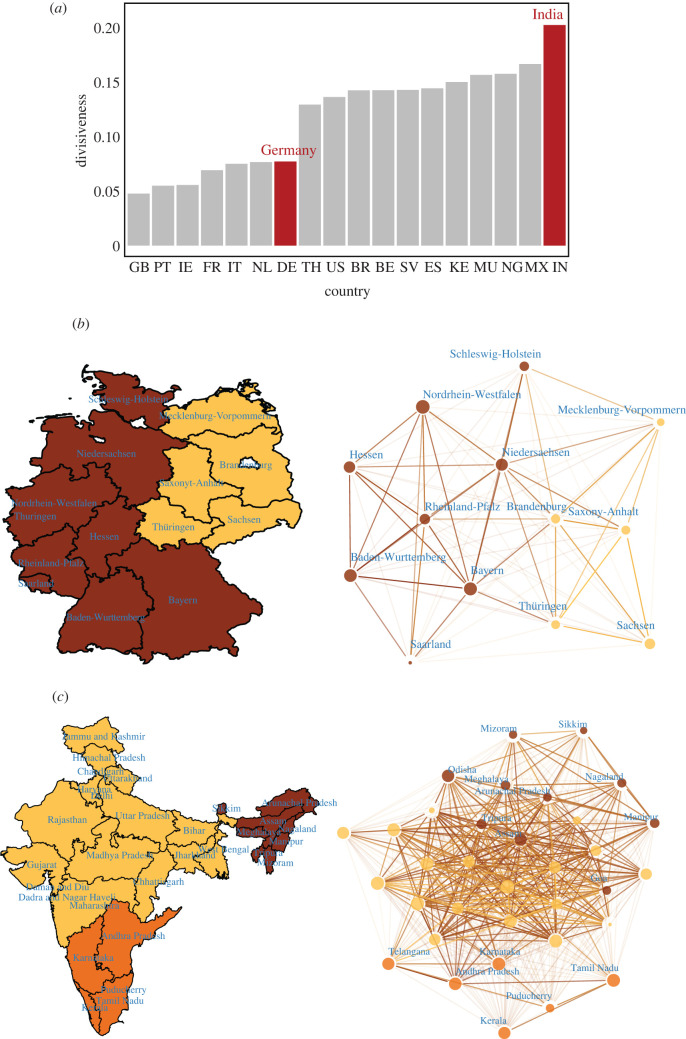
Regional divisiveness. (*a*) Population-weighted regional divisiveness for 18 countries (range for number of regions: [3,76]). Countries included are: BE, Belgium; BR, Brazil; DE, Germany; ES, Spain; FR: France; GB, Great Britain; IE, Ireland; IN, India; IT, Italy; KE, Kenya; MU, Mauritius; MX, Mexico; NG, Nigeria; NL, Netherlands; PT, Portugal; SV, El Salvador; TH, Thailand; US, United States. We chose these 18 countries to have a mix of developed countries and developing countries, as well as a mix of large and small countries. (*b*) Geographical map and network of regions in Germany (13 regions). Two communities of regions are detected. (*c*) Geographical map and network of regions in India (34 regions). Three communities of regions are detected. Networks are constructed from nodes as regions, and links are weighted by standardized cosine similarity between regions. Communities are detected using a multi-level modularity optimization algorithm (Louvain method) [79]. Nodes are resized proportionally to region population, and are coloured according to community affiliation. Links are coloured according to adjacent nodes, with lower transparency applied to higher weight links. Map regions are coloured according to communities calculated from the corresponding network. (*a*) Population-weighted regional divisiveness, (*b*) Germany, (*c*) India.

Figure 4 demonstrates that regions within countries can bear substantial similarity to one another. However, not all countries are likely to have the same amount of within-nation cultural similarity. Do some countries have more regional cultural variation within them than do others? Figure 6 examines countries according to their interregional cultural divisiveness, or the average cultural distance between regions within a country (see electronic supplementary material, appendix A). Figure 6*a* ranks 18 selected countries in increasing order of interregional divisiveness. Two findings stand out in figure 6*a*. First, developed countries exhibit smaller interregional divisiveness when compared with developing countries, suggesting that they benefit from greater cohesiveness between regions. Second, within the group of developed countries, the three countries with the least interregional cohesiveness are Belgium, Spain and the USA. The first two have well-known regional issues, with threats of secession, whereas the third is a large geographical nation.

Figure 6*b*,*c* displays the geographical maps and networks of regions in Germany (13 regions) and regions in India (34 regions), respectively (see electronic supplementary material, appendix A). We detect two communities of regions in Germany that map closely to the historical east–west divide in the country, suggesting this cultural divide still persists to this day. We detect three regional communities in India that correspond roughly geographically with linguistic regions defined by the language families spoken within the country.

## 5. Other applications of Facebook-based distances

**Figure 7.**
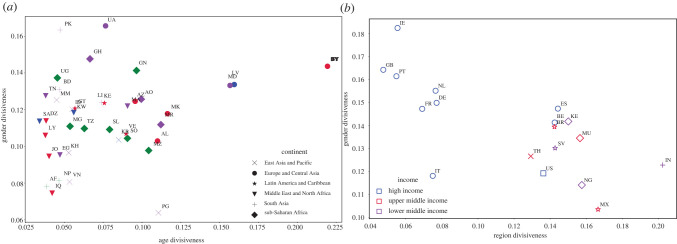
Subnational age, gender and regional cultural differences. (*a*) A scatter plot of age and gender divisiveness for 40 countries. The gender divisiveness is computed as the cosine similarity between the FB penetration vector for women and men using our sample of Facebook interests. The age divisiveness is computed as the median cosine similarity between the Facebook culture vector of three different age groups 15–29, 30–64 and 65+. (AF, Afghanistan; AL, Albania; AO, Angola; AZ, Azerbaijan; BD, Bangladesh; BY, Belarus; DZ, Algeria; EG, Egypt; GH, Ghana; GN, Guinea; GT, Guatemala; ID, Indonesia; IQ- Iraq; JO, Jordan; KH, Cambodia; KR, South Korea; KW, Kuwait; LK, Sri Lanka; LV, Latvia; LY, Libya; MA, Morocco; MD, Moldova; MG, Madagascar; MK, Macedonia; MM, Myanmar; MR, Mauritania; MZ, Mozambique; NP, Nepal; PE, Peru; PG, Papua New Guinea; PK, Pakistan; SA, Saudi Arabia; SL, Sierra Leone; SO, Somalia; TN, Tunisia; TZ, Tanzania; UA, Ukraine; UG, Uganda; VE, Venezuela; VN, Vietnam). Panel B shows a scatter plot of regional and gender divisiveness for 18 countries using (BE, Belgium; BR, Brazil; DE, Germany; ES, Spain; FR, France; GB, Great Britain; IE, Ireland; IN, India; IT, Italy; KE, Kenya; MU, Mauritius; MX, Mexico; NG, Nigeria; NL, Netherlands; PT, Portugal; SV, El Salvador; TH, Thailand; US, United States). (*a*) Divisiveness by age versus gender, (*b*) divisiveness by regions versus gender.

Figures 4–6 highlight the utility of our measure in assessing subnational questions that are simply too expensive to measure with traditional quantitative approaches. Yet differences in a society are not limited only to subnational differences. Societies can also differ along other identity cleavages, such as age, gender or race. Our measure enables us to also delve into the nature of cultural differences that vary according to such demographic groups and into differences that occur at even finer degrees of spatial resolution. Figure 7*a*,*b* explore whether countries that exhibit more divisiveness in one dimension also do so in other dimensions. It shows that age divisions and gender divisions have a weak positive association (Pearson correlation coefficient: 0.234, *p*: 0.146), whereas countries that suffer from greater regional divisions have smaller differences between men and women (Pearson correlation coefficient: −0.702, *p*: 0.001). Many developing countries exhibit more cohesiveness between genders, although they experience larger regional divides, compared to many developed countries.

**Figure 8.**
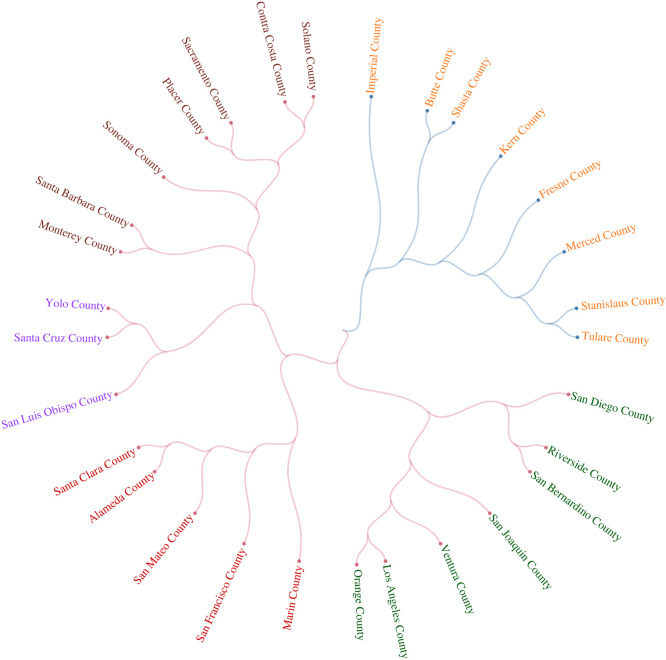
Local cultural similarities of California counties. The figure represents a dendrogram for California counties, based on cosine distances and Ward linkage method. In this figure, we examine interests with at least 20 000 users in the state, and in counties with a population of more than 75 000 users. Our unsupervised clustering returns clusters that are culturally sensible. The colour of a county’s link represents its membership of a main cluster, while the colour of its name represents its membership of a sub-cluster. Los Angeles County is closest to Orange County, Tulare County is most similar to Stanislaus County, Santa Cruz County is proximal to San Luis Obispo County and San Francisco County is nearest Marin County.

Furthermore, our data enable us to investigate cultural similarities and differences at even higher spatial resolution. Figure 8 depicts the dendrogram of the cultural clustering of the most populous California counties. Geographically disparate but culturally similar counties—such as coastal surfing communities of San Luis Obispo and Santa Cruz as well as the rural inland counties of Imperial and Butte—are located next to one another in the dendrogram. Generalizing this approach would allow us to identify which local areas culturally diverge from the rest of the nation in which they are located, a phenomenon that might provide insight into regional political disaffection.

## 6. Further discussion and concluding remarks

Our method lays out a complementary approach to the study of culture that can increasingly be measured via the application of computational social science to the ever larger portion of human lives that are unobtrusively and observationally measured online and offline. Doing so with our sample of Facebook interests for countries and subnational and local regions around the world indicates that our bottom-up measure of culture corresponds positively to traditional quantitative measures, contains a greater number of explanatory dimensions, enables the clustering of countries, subnational units, and localities into sensible groupings, and provides insight into cultural variation at unprecedented spatial, demographic and topic-based resolution. A further strength of our approach is its ability to answer questions about human culture that have been—up to this point—impossible to investigate at scale using traditional quantitative methods. For example, our method and data can enable us to investigate questions such as, ‘Which country is the cultural centre of the world?’ and ‘Which is the global ‘sister region’ of a particular region within a country?’ (see electronic supplementary material, appendix B4 and B6).

The high spatial (zip code-level) and temporal (daily) resolution of Facebook’s available data, coupled with the more than 200 million individuals on the platform in the United States and the more than two billion on the platform around the world, enable the measuring of cultural differences with remarkable precision. Computing cultural differences between subnational regions (figures 4–6), cities, counties (figure 8), or any different subgroups of any country (figure 7), which is cost-prohibitive when using traditional surveys, now becomes a straightforward endeavour. These studies can be conducted freely via the publicly available data provided by the Facebook Marketing API. Even so, this API is limited relative to what is theoretically possible, given the magnitude of human behavioural data that firms are currently collecting globally.

While we believe our conception and measurement of culture provide numerous complementary benefits to traditional measures, a number of considerations are worth noting. For instance, the fact that our approach does not inductively distill culture into parsimonious concepts means that the constellations of interests and behaviours that might diverge between two cultures may not always lend themselves to ease of conceptual interpretation. This is one drawback of our methodology.

Furthermore, while our Facebook measure of culture represents a marked improvement in terms of its ability to measure the surface of culture as compared to traditional surveys with high rates of non-response and relatively few questions [54], it is still far from perfect. Not all individuals in every country around the world are on Facebook. Our analysis only generalizes to differences among those who use Facebook (however, our validity results persist even when looking at countries with lower penetration of Facebook use; see electronic supplementary material, appendix B2). Additionally, while nearly 60 000 dimensions represent a dramatic increase over traditional top-down methods (of three orders of magnitude; see figure 2*a*), they still measure only a fraction of the full dimensionality of culture. Moreover, these dimensions themselves suffer from a certain endogeneity of measurement: Facebook does not have an interest listed for every possible feature of culture. Those it chooses to classify are endogenous to the platform itself. For example, these interests exclude certain topics—like sex and hate speech—which are banned from the platform. A more ideal computational system would classify all interests at an even finer scale of demarcation.

Finally, while we believe that our measure possesses numerous complementary benefits to traditional quantitative and qualitative approaches to culture—unobtrusiveness, scale, resolution, richness and breadth of constructs, and the ability to freely peer into the lives of billions of people—our measure emphatically cannot substitute for traditionally employed approaches to culture [80]. If we observe that individuals in a place spend substantial time looking at religious websites, we cannot know to what degree they personally hold religion as important: traditional approaches are needed to investigate further. And while our method is useful in providing a culturomic [81,82] barcoding [83,84] of global culture, the classification and interpretation of culturally important factors still necessitate in-depth and careful study of features uncovered by our approach—as well as of those features of culture that our measure omits.

Humans around the world share many cultural similarities but also have many differences. Until very recently, quantitatively measuring vast portions of culture was impossible. However, as we collect data on humanity it becomes increasingly possible to measure the surface of human culture in manners that approach cultures’ full underlying dimensionality. Doing so—in addition to furthering traditional quantitative and qualitative approaches to culture—will aid in a richer understanding of global human culture.

This improved ability to measure cultural differences between population groups at a more granular level enables a dramatic advance in the evaluation of some of the most pressing questions in the social sciences, such as: do national borders shape cultures? Are societies more likely to fracture along gender lines, racial lines, or regional lines? Which specific locations on the globe are more prone to civil conflict and violence? Does a lack of cultural cohesiveness contribute to political extremism? Are certain immigrant cultures more adept at integrating than others? Some of these questions have been touched upon in this paper; others have not. All have one element in common: answering them requires cultural measurement at previously unavailable scales and resolutions.

## Data Availability

Data and code will be available at the paper’s repository on OSF (http://doi.org/10.17605/OSF.IO/A2BTR) and in a public repository in Github (https://github.com/measuring-culture). The data description is provided in electronic supplementary material [85].
